# Evaluation of safety of preoperative GLP-1 receptor agonists in patients undergoing elective surgery: a systematic review, meta-analysis and meta-regression

**DOI:** 10.1016/j.eclinm.2025.103408

**Published:** 2025-08-12

**Authors:** Sivesh K. Kamarajah, Nadia Gudiozzi, John M. Findlay, Matthew J. Lee, Thomas Pinkney, Sheraz R. Markar

**Affiliations:** a Department of Applied Health Sciences, School of Health Sciences, College of Medicine and Health, University of Birmingham, Birmingham, UK; b Nuffield Department of Surgery, University of Oxford, Oxford, UK; c Department of Surgery, Churchill Hospital, Oxford University Hospitals NHS Trust, Oxford, UK; d Academic Department of Abdominal Wall Surgery, North Devon District Hospital, Royal Devon University Healthcare NHS Foundation Trust, Barnstaple, UK; e Department of Clinical and Biomedical Sciences, University of Exeter Medical School, Exeter, UK; f NIHR Exeter Biomedical Research Centre, Exeter, UK

**Keywords:** GLP1, Safety, Surgical systems, Evidence synthesis

## Abstract

**Background:**

Obesity remains a major global public health challenge, particularly among surgical patients, where it can be associated with increased perioperative and longer-term risk. While preoperative weight management strategies are often used to mitigate these risks, scalable interventions remain limited. Glucagon-like peptide-1 receptor agonists (GLP-1 RA) are an emerging pharmacological approach for weight loss, but their perioperative safety remains uncertain. This study aimed to assess the safety and efficacy of preoperative GLP-1 therapy in elective surgical systems.

**Methods:**

In this systematic review and meta-analysis, we systematically searched PubMed, MEDLINE, Embase, and the Cochrane Library from database inception to March 31, 2025, for studies evaluating preoperative GLP-1 RA use in adults undergoing elective surgery, with no language restriction. The primary outcome was perioperative safety, defined as any complication within 90 days after surgery. The secondary outcome was preoperative weight loss. Both frequentist and Bayesian random-effects meta-analyses were conducted. A Bayesian hierarchical meta-regression was used to explore effect modifiers on the primary outcome. A risk-of-bias and Grading of Recommendations, Assessment, Development, and Evaluation (GRADE) assessment were done to determine the certainty of the evidence. Final GRADE judgements were made by two independent reviewers, with consensus reached through discussion. Between-study heterogeneity was quantified using the *I*^*2*^ statistic and visualised using a standard forest plot. Potential publication bias and small study effects were assessed using visual inspection of funnel plots and Egger's test. This study is registered with PROSPERO, CRD420251027809.

**Findings:**

A total of 21 studies, comprising 97,059 patients, met inclusion criteria; 31.9% (n = 30,981) received preoperative GLP-1 RA therapy. Most studies were single-centre observational cohorts from high-income countries, with no randomised trials identified. Postoperative complications were reported in 12 studies, with no evidence of increased risk in GLP-1 users (pooled odds ratio: 0.78, 95% confidence interval: 0.59–1.05). The pooled estimate shows high heterogeneity (*I*^*2*^ = 73%). Bayesian meta-analysis yielded consistent findings (posterior mean OR: 0.78; 95% credible interval: 0.57–1.12). Meta-regression identified no statistically significant modifiers of treatment effect to explain the heterogeneity. The overall GRADE assessment for certainty of evidence was very low. In studies reporting weight loss, preoperative GLP-1 RA use was associated with weight loss of up to 16.7 kg or 6.0 kg/m^2^ over six months, though reporting varied across studies.

**Interpretation:**

Preoperative GLP-1 RA therapy may support clinically meaningful weight loss without a clear signal of increased perioperative risk, offering a potentially scalable strategy for surgical optimisation. However, the certainty of current evidence is very low, with most studies observational in design and at risk of bias. Amid rising global obesity rates and mounting surgical backlogs, the role of GLP-1RAs in perioperative care remains a critical unanswered question. Robust randomised trials are needed to establish their clinical value, cost-effectiveness, and implementation potential across diverse surgical systems. Prioritisation by funders and policymakers are needed as part of broader health policy agendas to improve population health and health system resilience.

**Funding:**

10.13039/501100000272NIHR Doctoral Research Fellowship and 10.13039/100014461NIHR Exeter Biomedical Research Centre Senior Investigator Fellowship.

**Research in context**

**Evidence before this study**

There is growing evidence from randomised controlled trials that GLP-1 receptor agonists are associated with clinically significant weight loss in individuals with overweight or obesity. However, their safety in patients undergoing elective surgery remains uncertain. We performed a systematic search of PubMed, MEDLINE, Embase, and the Cochrane Library from database inception to March 31, 2025, using the terms “GLP-1” OR “glucagon-like peptide-1 receptor agonist” AND (“surgery” OR “perioperative” OR “preoperative”), with no language restrictions. We identified no prior systematic review, or meta-analysis had comprehensively examined whether GLP-1 therapy could be safely integrated into preoperative pathways, revealing a critical knowledge gap.

**Added value of this study**

To our knowledge, this is the first systematic review, meta-analysis, and meta-regression to consolidate and critically appraise evidence regarding perioperative safety in patients receiving preoperative GLP-1 therapy. By analysing data from more than 97,000 patients undergoing elective surgery across 21 studies, our findings provide a clearer picture; there is a lack of high quality and randomised studies, but GLP-1 receptor agonists appear to effect clinically meaningful weight loss before elective surgery, without evidence of adverse perioperative outcomes. This suggests that elective surgery undertaken in patients receiving pre-operative GLP-1 therapy is safe, although the true effect on perioperative outcomes and longer term outcomes remains unclear. Together, preoperative GLP-1 may offer a novel avenue for optimising patients with obesity prior to surgery and addresses an unfilled knowledge gap concerning perioperative safety and efficacy of these agents.

**Implications of all the available evidence**

Undertaking elective surgery following pre-operative treatment with GLP-1 receptor agonists appears safe and an effective intervention to induce weight loss in patients with overweight or obesity. This suggests GLP-1 receptor agonists are a candidate for a scalable pharmacological strategy to reduce weight before surgery and potentially improve perioperative and longer term outcomes. However, the current evidence base is predominantly observational, with little standardisation in safety reporting. High-quality randomised trials are required to validate these findings, establish cost-effectiveness, and clarify implementation pathways. Addressing these gaps is crucial for informing global health policy, improving perioperative care standards, and harnessing the full potential of GLP-1 therapies to reduce the burden of obesity in surgical populations worldwide. As next steps, designing platform randomised trials to understand clinical- and cost-effectiveness of GLP-1 RA in surgical pathways are needed.

## Introduction

Obesity is a rising global epidemic, with its prevalence tripling over the past four decades.^1^^,^^2^ It is now recognised as a major driver for developing long-term conditions or multimorbidity,^3^^,^^4^ increasing the risk of cardiovascular disease, type 2 diabetes mellitus (T2DM), and several cancers.^5^ Excess weight also predisposes individuals to metabolic dysfunction, chronic inflammation, and impaired immune responses, all of which have substantial implications upon health outcomes.^5^ Interventions to facilitate weight loss in individuals with overweight or obesity have been challenging.^6^ These challenges arise from variable levels of implementation due to shortage of workforce such as dieticians, lack of perceived benefits from available interventions and absence of robust budget impact modelling on the benefits.^7^ However, more recently, glucagon-like peptide-1 receptor agonists (GLP-1 RA) have emerged as a new pharmacological intervention,^8^^,^^9^ initially developed for T2DM but now transforming the management of obesity. Pharmacological agents such as liraglutide and semaglutide have demonstrated efficacy in weight loss,^10^ offering an alternative to more invasive interventions, and shifting the paradigm of non-surgical obesity treatment from behavioural strategies to targeted effective medical therapy.

With 313 million people undergoing surgery each year around the world,^11^^,^^12^ obesity is particularly prevalent among surgical patients, with implications that extend beyond the direct metabolic burden. Excess adiposity is associated with increased perioperative risk, including anaesthetic complications, delayed wound healing, increased infection rates, and prolonged hospital stays.^13^ Traditional non-surgical weight loss interventions, such as lifestyle modification and funded pathways within hospitals and the community, have shown variable success in optimising patients for surgery. Therefore, there remains a clinical need for effective, scalable, and preoperatively applicable strategies.^14^^,^^15^ Given their profound impact on weight reduction and metabolic control, GLP-1 RAs have attracted increasing interest as a preoperative intervention to improve surgical outcomes. Despite this, there remains a knowledge gap regarding the safety and weight loss profiles in patients undergoing surgery.

To better understand this knowledge gap, the aim of this systematic review was to appraise the existing literature on the pre-operative use of GLP-1 RAs in elective surgery. Second, we aimed to establish the perioperative safety and understand the weight loss profiles associated with preoperative GLP-1 RA therapy.

## Methods

### Study design and ethics

This was a prospectively registered systematic review (PROSPERO: CRD420251027809) and reported according to the Preferred Extension for Systematic Review (PRISMA)^16^ guidelines (Appendix p 2–4, Table S1). This scoping review was conducted according to the Cochrane Training for Systematic Reviews.^17^ For this systematic review, the research question was: *What is the existing evidence on the preoperative use of GLP-1 receptor agonists patients undergoing surgery?* This review aimed to identify relevant studies evaluating its preoperative weight loss and safety of the GLP-1's when used in the perioperative setting. Ethical approval and informed consent was not applicable for this study, as this was a systematic review of existing literature and did not involve direct contact with human participants.

### Selection criteria and search strategy

We developed a systematic search strategy to identify relevant literature. Systematic searches were performed in MEDLINE (Ovid), Embase (Ovid), PsycINFO (Ovid), CINAHL Plus (EBSCO), and Cochrane Library to identify relevant studies from inception to 31st March 2025. The systematic search was carried out on the 3rd April 2025. The search strategy combined Medical Subject Headings (MeSH) and free-text terms related to “GLP-1” OR “GLP-1 receptor agonists” AND “preoperative” OR “surgery”. No date restrictions were applied, but only peer-reviewed articles published in English were included. The full search strategy, developed with an information specialist, is shown in the Appendix (p 5, Table S2). To supplement the database search, we hand-searched reference lists of any clinical practice guidelines that we found, to maximise capture of relevant guidelines.^18^ For this systematic review, the inclusion criteria were: (i) studies evaluating GLP-1 RA in adult (≥18 years old) surgical patients; (ii) studies assessing weight reduction, metabolic outcomes, or perioperative complications; and (iii) any study design such as randomised controlled trials, cohort studies, or case-control studies. Exclusion criteria were: (i) case reports, reviews, and editorials with no patient-level data; (ii) studies where GLP-1 RA were used exclusively postoperatively; and (iii) non-human or in vitro studies.

### Study selection and data extraction

All studies identified in the scoping review were extracted into Microsoft Excel. The study screening was delivered over a two-staged process by two independent reviewers (SKK, NG). In stage 1, all the titles and abstracts were screened by two authors (SKK, NG). Disagreements were resolved by consensus, and in the case that consensus could not be reached, the senior author (SRM) arbitrated. Once agreement was reached on the included studies, the titles and abstracts were screened again by two more authors, prior to retrieving full texts. Data were extracted from eligible studies using a structured extraction form and presented in the Appendix (p 6). Extracted variables included study characteristics (author, year, study design), patient demographics, type and duration of GLP-1 therapy, surgical procedure, primary and secondary outcomes, and reported adverse events. Where applicable, quantitative data on weight loss, glycaemic control, and perioperative outcomes were extracted for synthesis. Two reviewers (SKK, NG) independently performed data extraction, and discrepancies were resolved by consensus.

### Outcome measures

There is currently no universally accepted core outcome set for evaluating the perioperative safety of GLP-1 RA in patients undergoing surgery. Therefore, the primary outcome was perioperative safety, operationalised as the incidence of any postoperative complication occurring within 90 days of surgery. Postoperative complications were considered broadly, including both surgical and medical events, defined according to the Clavien–Dindo classification.^19^^,^^20^ This classification is a widely adopted and validated system that grades the severity of complications based on the type of therapeutic intervention required. Two key secondary outcomes were preoperative weight loss and GLP-1-specific adverse events. Preoperative weight loss was defined as the absolute or percentage change in body weight from initiation of GLP-1 therapy to the time of surgery. Adverse events from GLP-1 were defined as aspiration pneumonia, delayed gastric emptying or gastroparesis, hypoglycaemia (in patients with or without concurrent insulin use), postoperative nausea and vomiting, and intraoperative or postoperative haemodynamic instability attributed to GLP-1 use.

### Overview of analysis plan

We adopted a staged analytic approach, using both frequentist and Bayesian approaches, to provide a robust understanding on the influence of preoperative GLP-1 on perioperative safety. *Primary:* Estimate the pooled effect of the preoperative GLP-1 on perioperative safety, measured by postoperative complications, up to 90-days from surgery. *Secondary:* Understand the influence of study modifiers on treatment effect estimates.

### Statistical analysis

Descriptive data were tabulated to summarise study characteristics, including setting, population, intervention and comparator groups, outcomes, and methodological features. Continuous variables were summarised using means and standard deviations (or medians and interquartile ranges, as appropriate), and categorical variables using frequencies and percentages. Clinical and methodological heterogeneity were assessed qualitatively to support decisions regarding meta-analysis. Effect estimates were extracted or calculated as odds ratios (ORs) with associated 95% confidence intervals (CIs). The natural logarithm of the OR and its standard error were used for all statistical modelling. All available studies were included in the primary meta-analysis regardless of missing covariate data. For the meta-regression models, only studies with complete information on all four modifiers were included. We did not impute missing covariate values, given the limited number of studies and the risk of introducing additional uncertainty. All statistical analyses were conducted using R (version 3.2.2) with ggplot2, tidyverse, rjags, and metafor, packages (R Foundation for Statistical Computing, Vienna, Austria).

For the primary analysis, we first conducted a random-effects meta-analysis using restricted maximum likelihood estimation (REML). Non-comparative studies reporting single-arm data were excluded from the primary meta-analysis of postoperative complications, which pooled measures of association between patients receiving GLP-1 receptor agonists and those not receiving them. Data from non-comparative studies were summarised narratively for descriptive outcomes such as weight loss and adverse events. The DerSimonian–Laird estimator was not used due to known limitations with small samples or heterogeneity; REML provides a more robust estimate of between-study variance. This generated a pooled OR and its 95% CI. Between-study heterogeneity was quantified using the *I*^*2*^ statistic and visualised using a standard forest plot. As a sensitivity analysis, we conducted a Bayesian random-effects meta-analysis on the same studies. This model was specified on the log OR scale and included a global treatment effect parameter and a variance parameter representing between-study heterogeneity. Non-informative (flat) priors were used for all parameters to minimise the influence of prior assumptions. The model was implemented in JAGS via the rjags package in *R*. Four Markov chains were run for 20,000 iterations each, with a burn-in of 5000 iterations and thinning interval of 20. Convergence was assessed using trace plots and the Gelman–Rubin diagnostic statistic. For the secondary analysis, we conducted a meta-regression including the pre-specified modifiers. The modifiers of interest were mean age, proportion of female patients, mean body mass index (BMI), and the proportion of patients with diabetes in the study population. These variables were selected a priori based on clinical relevance across published studies. We conducted a hierarchical Bayesian meta-regression model incorporating the same study-level modifiers, as well as a country-level random intercept to account for contextual variation. This three-level model specified the log OR for each study as a linear combination of the modifiers, plus a random effect for the country in which the study was conducted. All parameters were assigned non-informative priors, Gaussian priors, with large variances for regression coefficients (i.e., weakly informative), and inverse gamma priors for variance parameters. Model fitting and convergence procedures mirrored those used in the primary Bayesian meta-analysis. Posterior means and 95% credible intervals were summarised for all model parameters. Predicted treatment effects across the range of each continuous modifier (i.e., age, BMI) were plotted while holding other covariates at their mean values. Forest plots were used to compare observed and posterior study-level treatment effects.

### Assessing certainty of evidence

We assessed the certainty of evidence using the Grading of Recommendations Assessment, Development and Evaluation (GRADE) approach. Certainty ratings were applied to the pooled effect estimates for each outcome and categorised as high, moderate, low, or very low, based on five domains: (i) risk of bias; (ii) inconsistency; (iii) indirectness; (iv) imprecision; and (v) publication bias. The certainty of evidence from randomised trials starts at high, and non-randomised trials start at low. Direct evidence can be rated down for risk of bias, inconsistency, indirectness, or small study effects.^21^ Risk of bias was evaluated using the ROBINS-I tool for observational studies and RoB-2 for the one included randomised trial (Appendix p 14). For inconsistency, we evaluated heterogeneity using the *I*^*2*^ statistic, random-effects modelling, and explored study-level modifiers via Bayesian hierarchical meta-regression as described above. We rated down for imprecision when 95% CI included both the null and clinically meaningful effects, or when event counts were low relative to the optimal information size. Potential publication bias and small study effects were assessed using visual inspection of funnel plots and Egger's test. Final GRADE judgements were made by two independent reviewers, with consensus reached through discussion.

### Role of the funding source

The funders had no role in study design, data collection, data analysis, data interpretation, writing of the report, or the decision to submit the paper for publication.

## Results

### Literature search

**Fig. 1:**
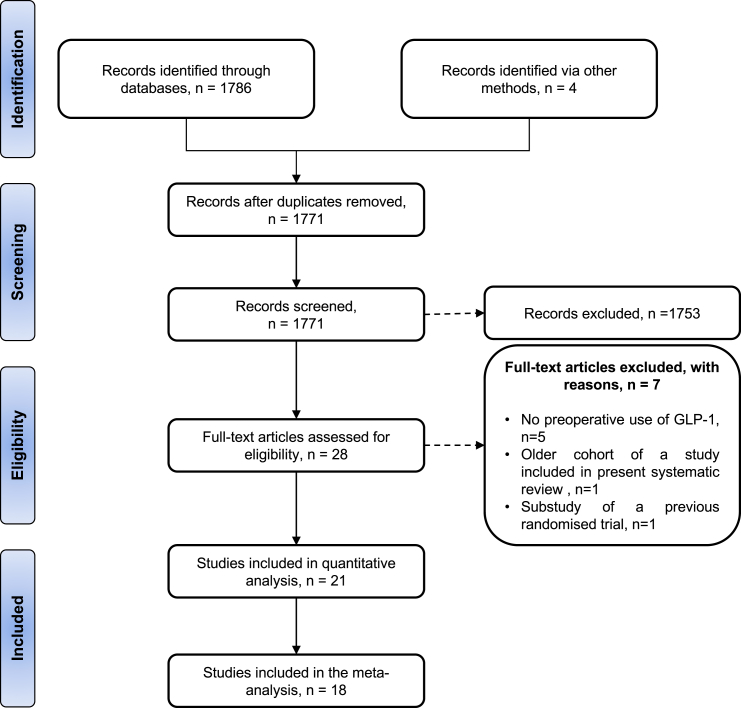
PRISMA flow diagram of systematic review.

This review identified 1801 studies. Of the 28 studies that underwent full text review, five studies did not evaluate pre-operative GLP-1, one was an older cohort of a study included in the present systematic review, and one study was a sub-study of a previously published randomised controlled trial, leading to seven exclusions in total. Finally, 21 studies^22^, ^23^, ^24^, ^25^, ^26^, ^27^, ^28^, ^29^, ^30^, ^31^, ^32^, ^33^, ^34^, ^35^, ^36^, ^37^, ^38^, ^39^, ^40^, ^41^, ^42^ were included that met the inclusion criteria (Table 1). Fig. 1 presents the PRISMA flowchart documenting the study selection process.

**Table 1:** Study characteristics reporting perioperative use GLP-1.

Study name	Study duration	Country	Number of centres	Patients, n	Comparative^a^
Wood 2016^24^	January 2004–February 2011	United States	Single centre	150	Yes
Tang 2017^23^	NR	United Kingdom	Single centre	45	No
Polderman 2018^22^	February 2014–January 2017	Netherlands	Multicentre	150	Yes
Hulst 2020^25^	June 2017–November 2018	Netherlands	Multicentre	278	Yes
Ilanga 2023^27^	October 2020–May 2022	United States	Single centre	31	Yes
Martines 2023^29^	January 2019–January 2022	Italy	Single centre	86	Yes
Dixit 2024^26^	January 2015–December 2021	United States	Multicentre	23,679	Yes
Gonzalez 2024^30^	August 2019–December 2023	United States	Single centre	19	No
Magruder 2024^42^	January 2010–October 2021	United States	Multicentre	9465	Yes
Martines 2024^28^	March 2017–October 2022	Italy	Single centre	180	Yes
Munoz 2024^31^	July 2022–December 2022	Mexico	Single centre	37	No
Oosterom-Eijmael 2024^32^	April 2018–August 2018	Netherlands	Single centre	25	Yes
Spurzem 2024^33^	2014–2023	United States	Single centre	46	Yes
Welk 2024^34^	February 2020–March 2023	Canada	Multicentre	17,905	Yes
Aschen 2025^41^	February 2020–July 2023	United States	Single centre	35,020	Yes
AbuHasan 2025^35^	2018–2023	United States	Multicentre	2169	Yes
Kim 2025^37^	January 2010–October 2022	United States	Multicentre	5950	Yes
Mathur 2025^38^	2017–2024	United States	Single centre	364	Yes
Rayman 2025^39^	February 2023–September 2023	Israel	Multicentre	434	Yes
Seddio 2025^40^	2010–2022	United States	Multicentre	904	Yes
Sen 2025^36^	June 2023–July 2023	United States	Single centre	124	Yes

a Non-comparative studies only included patients receiving preoperative GLP-1 agonists, with no comparator group.

### Study characteristics

Of the 21 studies included, 18 (78.3%) were published in the past five years. The majority (n = 18/21, 85.7%) were published from high income countries and eleven were published from the United States. Of the included studies, three studies were non-comparative (n = 101 patients) and these were single-centre retrospective cohort studies. Of the remaining 18 studies, two were multicentre randomised controlled trials, two were single centre prospective cohort studies, eight were single centre retrospective cohort studies and six were multicentre retrospective cohort studies. For the two randomised controlled trials, both were comparing GLP-1 receptor agonists (vs. placebo or standard of care).

### Patient characteristics

This scoping review included 97,059 patients, of which 31.9% (n = 30,981) received preoperative GLP-1. The type of GLP-1 medications used were reported in 16 studies, of which liraglutide and semaglutide were the most common. The most common type surgeries that patients underwent were bariatric (n = 11 studies). A summary of the patient characteristics of the included studies are presented in Table 2.

**Table 2:** Patient characteristics of included studies reporting perioperative use GLP-1.

Study name	Type of surgery	Type of GLP-1	Duration of preoperative GLP-1	Patients, n		Age, years^a^		Female, n (%)		Body mass index^a^, kg/m^2^		Diabetes, n (%)	
				With GLP-1	No GLP-1	With GLP-1	No GLP-1	With GLP-1	No GLP-1	With GLP-1	No GLP-1	With GLP-1	No GLP-1
Wood 2016^24^	Bariatric	GLP + No Insulin	NR	14	43	47.9 (7.8)	48.8 (10.7)	9 (64.3)	35 (81.4)	–	–	14 (100.0)	43 (100.0)
		GLP-1 & insulin	NR	23	68	53.2 (6.7)	53.7 (9.3)	15 (65.2)	42 (61.8)	–	–	23 (100.0)	68 (100.0)
Tang 2017^23^	Bariatric	Liraglutide (n = 35); Exenatide (n = 10)	NR	45	–	NR	–	NR	–	NR	–	NR	–
Polderman 2018^22^	Non-cardiac	Liraglutide	16 weeks	44	106	62.0 (8.8)	62.0 (8.1)	24 (54.5)	55 (51.9)	29.0 (26.0–33.0)	28.0 (26.0–35.0)	44 (100.0)	106 (100.0)
Hulst 2020^25^	Cardiac	Liraglutide	NR	139	139	64.6 (11.2)	65.3 (10.7)	24 (17.3)	26 (18.7)	27.3 (4.0)	27.7 (4.4)	21 (15.1)	21 (15.1)
Ilanga 2023^27^	Bariatric	NR	20 weeks	18	13	NR	NR	NR	NR	60.7 (6.6)	54.7 (3.8)	9 (50.0)	5 (38.5)
Martines 2023^29^	Bariatric	Liraglutide	24 weeks	42	44	41.0 (35.5–48.0)	41.5 (37.5–49.3)	39 (92.9)	34 (77.3)	57.5 (51.2–59.2)	55.9 (53.3–59.3)	35 (83.3)	38 (86.4)
Dixit 2024^26^	General surgery	NR	NR	3502	20,177	53.6 (8.1)	53.9 (8.9)	1720 (49.1)	10,994 (54.5)	–	–	3502 (100.0)	20,177 (100.0)
Gonzalez 2024^30^	Transplant	Liraglutide (n = 6); Semaglutide (n = 8); Tirzepatide (n = 1)	NR	19	–	NR	–	5 (26.3)	–	42.0 (34.6–48.8)	–	16 (84.2)	–
Magruder 2024^42^	Orthopaedic	Semaglutide	NR	1653	7812	NR	NR	787 (47.6)	3736 (47.8)	NR	NR	1540 (93.2)	7313 (93.6)
Martines 2024^28^	Bariatric	Liraglutide	24 weeks	87	93	41.0 (35.5–48.0)	43.0 (38.5–50.0)	65 (74.7)	74 (79.6)	42.2 (41.1–43.6)	45.0 (41.0–47.5)	42 (48.3)	46 (49.5)
Munoz 2024^31^	Bariatric	NR	12 weeks	37	–	44.0 (9.8)	–	28 (76.0)	–	56.0	–	9 (24.3)	–
Oosterom-Eijmael 2024^32^	Cardiac	Liraglutide	NR	13	12	61.9 (15.7)	62.4 (12.9)	2 (15.4)	2 (16.7)	26.7 (4.6)	27.3 (3.5)	2 (15.4)	1 (8.3)
Spurzem 2024^33^	General surgery	NR	6.3 (4.0) months	24	22	56.5 (11.6)	61.8 (11.1)	16 (66.7)	13 (59.1)	38.1 (4.9)	NR	NR	NR
Welk 2024^34^	Non-cardiac	Semaglutide	NR	3833	14,072	71 (68.0–75.0)	72 (69.0–75.0)	1499 (39.1)	5548 (39.4)	NR	NR	3833 (100.0)	14,072 (100.0)
AbuHasan 2025^35^	Bariatric	Semaglutide (n = 109); Dulaglutide (n = 77); Liraglutide (n = 71); Tirzepatide (n = 34); Exenatide (n = 2).	7.4 (3.9) months	293	1876	47.0 (38.0–54.0)	43.5 (36.0–53.0)	278 (94.9)	710 (37.8)	44.9 (40.8–50.7)	45.5 (41.8–51.0)	236 (80.5)	448 (23.9)
Aschen 2025^41^		NR	NR	17,510	17,510	64.0 (58.0–72.0)	65.0 (56.0–73.0)	9138 (52.2)	9064 (51.8)	28.0 (24.5–32.3)	31.1 (27.0–36.3)	17,510 (100.0)	17,510 (100.0)
Kim 2025^37^	Orthopaedic	Semaglutide; Dulaglutide; Liraglutide; Tirzepatide	NR	2975	2975	62.2 (7.6)	62.2 (7.6)	1986 (66.8)	1989 (66.9)	NR	NR	1571 (52.8)	1571 (52.8)
Mathur 2025^38^	Bariatric	Semaglutide	24 weeks	182	182	47.0 (36.0–56.0)	44.0 (33.0–56.0)	139 (76.0)	143 (79.0)	42.6 (39.2–48.6)	43.0 (39.1–47.9)	79 (43.0)	79 (43.0)
Rayman 2025^39^	Bariatric	Liraglutide (n = 168); Semaglutide (n = 155)	19 weeks	275	159	39.3 (12.7)	41.9 (12.7)	185 (67.3)	107 (67.3)	41.6 (6.2)	41.5 (6.1)	49 (17.8)	17 (10.7)
Sen 2025^36^	General surgery	Semaglutide (n = 39); Dulaglutide (n = 14); Tirzepatide (n = 9)	NR	62	62	59.0 (48.0–65.0)	53.0 (43.0–64.0)	37 (59.7)	38 (61.3)	33.3 (30.2–39.9)	34.2 (31.2–39.0)	44 (71.0)	14 (22.6)
Seddio 2025^40^	Neurosurgery	Semaglutide	NR	191	713	60.6 (8.5)	60.6 (8.2)	114 (59.7)	427 (59.9)	NR	NR	191 (100.0)	713 (100.0)

Abbreviation: NR-not reported.

a Age was presented as either mean with standard deviation or median with interquartile range.

### Postoperative complications

**Fig. 2:**
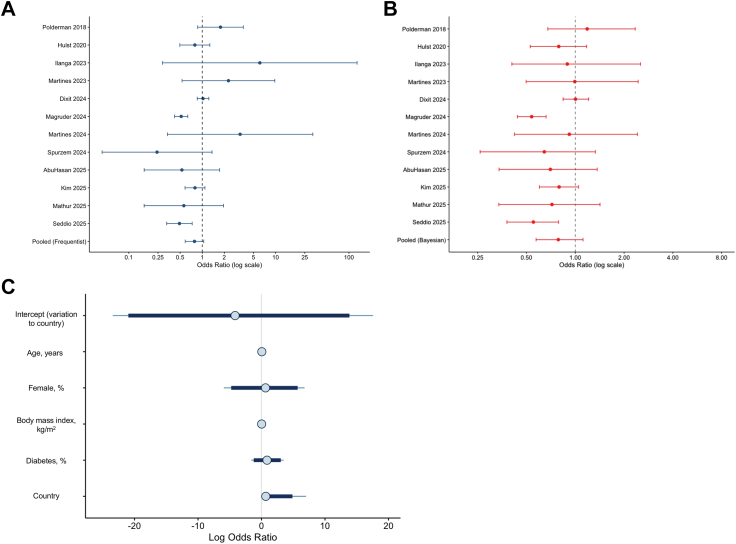
. Meta-analysis of association between GLP-1 on perioperative safety, measured using overall complications up to 90-days from surgery. (A) Random-effects frequentist meta-analysis. (B) Random-effects Bayesian meta-analysis as a sensitivity analysis with pooled odds ratio and 95% credible intervals. (C) Bayesian meta-regression modifiers, demonstrating no influence of study-level covariates on estimates, but strong correlation on the country-level effects. The intercept is variation due to country.

Of the 21 studies identified, twelve studies reported postoperative complications within 30-days from surgery. In a frequentist random-effects model, the pooled OR was 0.78 (95% CI: 0.59–1.05), with high between-study heterogeneity (*I*^*2*^ = 73.0%) (Fig. 2A). To assess the robustness of this finding, we conducted a Bayesian random-effects meta-analysis on the same dataset using non-informative priors. The posterior mean log OR was 0.86 (95% credible interval [CrI]: 0.61–1.22) (Fig. 2B), closely aligned with the frequentist estimate. The between-study heterogeneity, summarised by the posterior standard deviation of the random effects, was estimated at 0.36 (95% CrI: 0.11–0.84). These results suggest consistency in both the direction and magnitude of the pooled effect across modelling approaches. To explore potential sources of heterogeneity, we conducted a hierarchical Bayesian meta-regression including four pre-specified study-level modifiers, with a country-level random intercept included to account for contextual variation. Posterior mean estimates and 95% CrIs for each covariate indicated no statistically meaningful associations with treatment effect. All CrIs included the null, and effect sizes were small in magnitude. For instance, the estimated coefficient for diabetes prevalence was 1.53 (95% CrI: 1.14–4.08), suggesting a possible positive association, though with substantial uncertainty. Age, BMI, and proportion female showed no directional association. The between-country heterogeneity, reflected by the posterior standard deviation of the country-level random effect, was estimated at 2.17 (95% CrI: 0.10–12.93) (Fig. 2C), indicating potential variation in effect size by country. Although the credible interval was wide, the lower bound above zero supports the possibility of meaningful contextual heterogeneity.

**Fig. 3:**
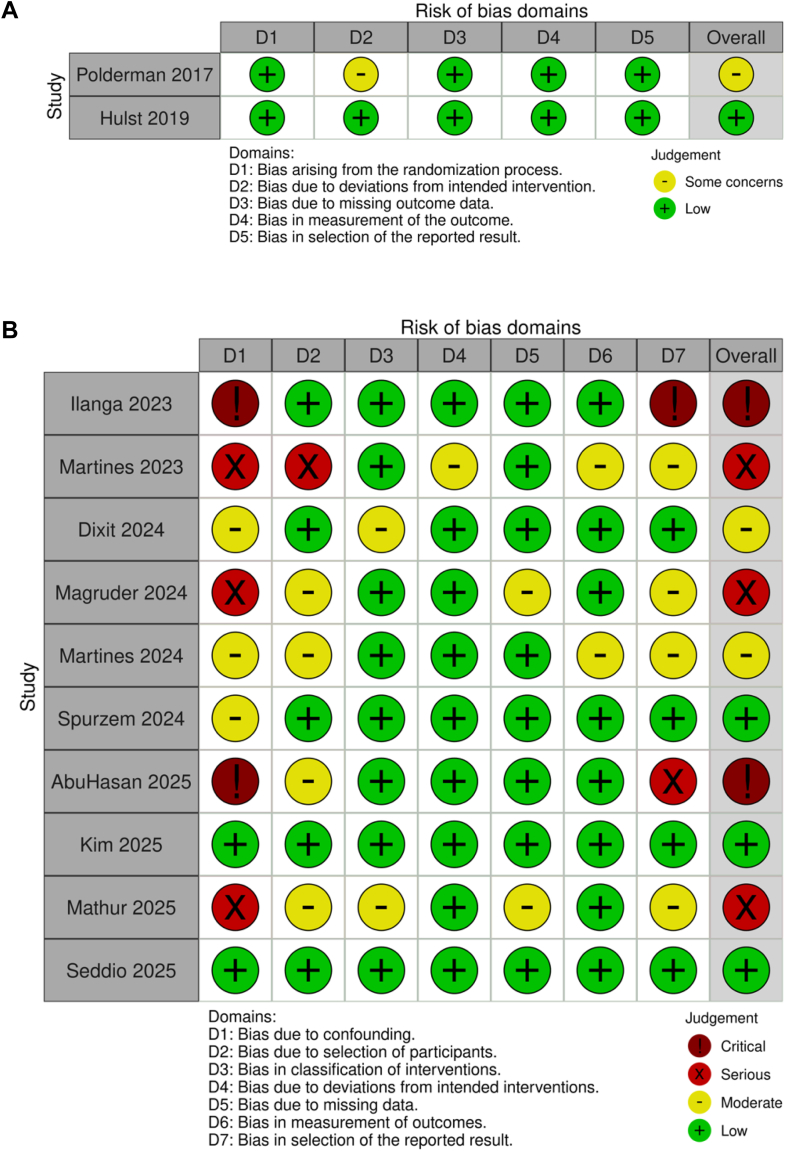
Risk of bias assessment for the studies included into the meta-analysis. (A) The Cochrane Risk of Bias-II tool was used to evaluate randomised studies and (B) ROBINS-I was used to evaluate non-randomised studies.

The certainty of evidence for the primary outcome, postoperative complications, was rated as very low. Although the pooled OR suggested a potential protective effect (OR 0.78 [95% CI: 0.59–1.05]), this result was imprecise and heterogeneous. Most included studies were observational and at moderate to serious risk of bias (Fig. 3, Appendix p 7–9). Bayesian meta-regression did not identify explanatory covariates for the observed heterogeneity. Publication bias was not detected on funnel plot or Egger's test (Appendix p 10; Figure S1). Nevertheless, due to residual risk of bias, substantial unexplained inconsistency, and imprecision, the overall certainty remained very low (Appendix p 11).

### Preoperative weight loss

Ten studies reported evaluation of preoperative weight loss before surgery using three different measures: (i) absolute body weight (n = 2); (ii) change in absolute body weight (n = 4); and (iii) proportion of excess body weight loss (n = 3) up to 52 weeks. The three studies reporting a change in body weight reported a reduction of weight up to 16.7 kg over six months, but the type of GLP-1 remains unclear. The two studies reporting change in body mass index reporting a reduction of up to 6.0 kg/m^2^ with use of GLP1 over 6 months. The four studies reporting proportion of excess body weight loss reported a reduction in weight up to 17%, but a preoperative treatment duration of 52 weeks.

### Adverse events from GLP-1

Only eleven studies reported safety or adverse events from the use of GLP-1 during the perioperative window. There were variable definitions used for adverse events. A summary of definitions and rates of adverse events are reported in Appendix (p 13). Only one study reported aspiration pneumonia, which showed no significant difference between patients with and without use of pre-operative GLP-1.

## Discussion

With the rapidly rising of prevalence of obesity around the world, particularly in surgical patients, identifying effective interventions to promote weight loss prior to surgery allows improved perioperative outcomes and long-term health. To our knowledge, this systematic review provides the first comprehensive appraisal and synthesis of evidence on the safety of preoperative GLP-1 in elective surgical systems. Preoperative GLP-1 RA therapy may support clinically meaningful weight loss without a clear signal of increased perioperative risk, offering a potentially scalable strategy for surgical optimisation. However, the certainty of current evidence is very low, with most studies observational in design and at risk of bias. Robust randomised trials are urgently needed to establish their clinical value, cost-effectiveness, and implementation potential across diverse surgical systems. Urgent prioritisation by funders and policymakers are needed as part of broader health policy agendas to improve population health and health system resilience.

To date, there have been growing concerns around safety of perioperative GLP-1,^43^ including the Pharmacovigilance Risk Assessment Committee of the European Medicines Agency.^44^^,^^45^ However, guidelines around this have lacked comprehensive evaluation of the literature on this topic. To our knowledge, this is the first evaluation summarising the evidence of the safety of preoperative GLP-1 in patients undergoing surgery. Some studies suggest an association between peri-operative GLP-1 RA use and a non-significant increased risk of pulmonary aspiration in the elective surgical setting.^46^ This is particularly important in the context of patients undergoing routine endoscopy.^47^ There are, however, conflicting data on the magnitude of these risks.^34^ In people using GLP-1 RAs long-term,^48^ there is less certainty in regard to the risk of pulmonary aspiration,^49^ particularly because the effect of tachyphylaxis on gastric emptying is unclear. Nevertheless, because of the action on slowing gastric emptying and early case reports of pulmonary aspiration, the American Society of Anesthesiologists (ASA) recommended these drugs should be stopped either the day before the procedure (for those on once-daily doses) or the week before (for those on weekly injections). This was, theoretically, to minimise the risk of incomplete gastric emptying leading to pulmonary aspiration on induction of anaesthesia.^50^ In its updated guidance, ASA recognises that the overall risk of perioperative complications appears low for most patients receiving GLP-1 RA, but continues to recommend precautionary withholding prior to procedures due to residual uncertainty.

There is supportive evidence supporting the benefits of weight loss prior to surgery.^51^^,^^52^ Preoperative weight loss has been associated with improved intraoperative technical performance, enhanced visualisation, and reduced operative time.^52^^,^^53^ Further, evidence from bariatric surgery cohorts suggests that weight loss before surgery is linked to better long-term outcomes, including lower postoperative complication rates, improved weight maintenance, and enhanced metabolic control.^53^^,^^54^ Despite these benefits, current strategies for achieving meaningful preoperative weight loss remain limited. First, very low-calorie diets (VLCDs) and structured meal replacements have been explored as alternatives to facilitate rapid weight reduction before surgery.^51^^,^^55^ VLCDs have shown promise in improving liver size and metabolic parameters preoperatively, which may enhance surgical safety and recovery. Second, supervised exercise regimens and behavioural modification programmes have been proposed as adjuncts to optimise weight loss and physical conditioning before surgery.^56^ Third, prehabilitation programmes incorporating lifestyle interventions, dietary modifications, and medical therapy have shown promise.^57^^,^^58^ Despite this, widespread implementation across health systems remains a challenge due to cost implications and variable adherence to these programs in real world clinical practice. Therefore, a multimodal approach combining dietary interventions, structured physical activity, and pharmacological agents such as GLP-1 RA may offer a more effective and scalable strategy for preoperative weight management.^59^

While the application of GLP-1 therapy in preoperative weight management is an emerging area, our findings align with randomised trials demonstrating significant weight loss with GLP-1 RA in non-surgical populations. The weight loss profiles observed in this review are consistent with those reported in large-scale RCTs, where GLP-1 therapies have achieved substantial reductions in body weight over comparable timeframes. For instance, the STEP trials evaluating semaglutide in non-surgical obesity have demonstrated weight reductions of up to 15% over 68 weeks.^2^ The findings from this review suggest that similar magnitudes of weight loss may be achievable in the preoperative period, though further prospective studies are required to confirm the clinical benefits in surgical patients. Notably, concerns have arisen around the potential impact of weight loss from GLP-1 prior to surgery. Some have expressed concern that whilst this might reduce obesity, it can have a negative impact on skeletal muscle, leading to sarcopenic obesity.^60^ Sarcopenia is a well-documented risk factor for surgical complications,^61^^,^^62^ and so pharmacological precipitation of this might be best avoided. Others have raised concerns about perioperative aspiration pneumonia.^63^ These considerations begin to highlight the unknowns of the interface of metabolic medicine and perioperative care.

Beyond weight loss, GLP-1 RA offer significant metabolic benefits, particularly in patients with type 2 diabetes.^6^^,^^9^ Research evidence demonstrates that GLP-1 therapy improves glycaemic control, reduces insulin resistance, and lowers cardiovascular risk factors. Randomised controlled trials such as the SUSTAIN and LEADER trials have shown that GLP-1 RA not only facilitate weight loss but also contribute to improved HbA1c levels and reduced incidence of major adverse cardiovascular events.^5^^,^^6^ In the context of surgery, optimising glycaemic control preoperatively is crucial, as hyperglycaemia has been associated with increased risk of infections, delayed wound healing, and prolonged hospital stays. This review identified two studies that evaluated the impact of GLP-1 therapy on glycaemic control in surgical patients, yet the heterogeneity in study designs limits definitive conclusions.^10^ Future studies should explore the dual role of GLP-1 RA in both weight reduction and metabolic optimisation to enhance perioperative care.

A key strength of this study is the systematic methodology employed, adherence to a pre-registered protocol, formal risk-of-bias assessments, and the use of GRADE to evaluate certainty of evidence on GLP-1 on postoperative complications. By including studies across various surgical specialities and intervention durations, this review provides a broad yet detailed overview of GLP-1's role in perioperative care. Additionally, this study highlights crucial gaps in the literature, particularly the lack of randomised controlled trials, emphasising the need for more rigorous investigations to establish definitive clinical recommendations. However, several limitations must be acknowledged. First, the heterogeneity of study designs, patient populations, and outcome measures limits direct comparisons across studies. The variability in weight loss assessment methods and the inconsistency in the definitions of postoperative complications pose challenges in drawing firm conclusions. Furthermore, the absence of standardised protocols for preoperative GLP-1 use reduces the generalisability of findings. Second, most included studies did not clearly report whether GLP-1 RA therapy was discontinued at the time of surgery or continued postoperatively, which raises the possibility that observed outcomes may reflect effects of continued perioperative use rather than strictly preoperative administration. Third, the field of medical obesity treatment is advancing rapidly, with newer agents such as tirzepatide, a dual GLP-1/GIP receptor agonist, now widely used in some countries. However, few studies to date have examined the perioperative safety profiles of these newer agents, which may differ from liraglutide or semaglutide. Future research should evaluate the safety and optimisation potential of these emerging therapies in surgical populations. Finally, as this review relied on published studies, there is a possibility of publication bias, where studies reporting positive outcomes may be overrepresented. In addition, as most included studies were single-centre observational cohorts from high-income countries, the generalisability of our findings to low- and middle-income settings is limited, highlighting the need for future research in more diverse global contexts. Future research should prioritise well-designed prospective trials with standardised endpoints to provide more definitive evidence on the role of GLP-1 therapy in the perioperative setting.

Preoperative GLP-1 RA therapy may support clinically meaningful weight loss without a clear signal of increased perioperative risk, offering a potentially scalable strategy for surgical optimisation. However, the certainty of current evidence is very low, with most studies observational in design and at risk of bias. Amid rising global obesity rates and mounting surgical backlogs, the role of GLP-1RAs in perioperative care remains a critical unanswered question. Robust randomised trials are urgently needed to establish their clinical value, cost-effectiveness, and implementation potential across diverse surgical systems. Urgent prioritisation by funders and policymakers are needed as part of broader health policy agendas to improve population health and health system resilience.

## Contributors

SKK, NG, and SRM contributed to data curation and interpretation. SKK and SRM contributed to data analysis. The writing group contributed to writing and critical revision of the manuscript. SKK and SRM accessed and verified the underlying data in the study. The corresponding author had final responsibility for the decision to submit for publication.

## Data sharing statement

Data sharing requests will be considered by the writing group upon written request to the corresponding author. De-identified participant data and/or other pre-specified data will be available, subject to a written proposal and an agreed data sharing agreement.

## Declaration of interests

We declare no competing interests.
