# Supplementary material for: Evaluation of safety of preoperative GLP-1 receptor agonists in patients undergoing elective surgery: a systematic review, meta-analysis and meta-regression
Source: eClinicalMedicine. 2025 Aug 12;87:103408. doi: 10.1016/j.eclinm.2025.103408 (PMC12496209; doi:10.1016/j.eclinm.2025.103408)
Supplement: Appendix–Tables and Figures [file mmc1.docx]

**Evaluation of safety of preoperative GLP-1 receptor agonists in patients undergoing elective surgery: A systematic review, meta-analysis and meta-regression.**

Sivesh K Kamarajah^1^, Nadia Gudiozzi^2, 3^, John M Findlay^4, 5, 6^, Matthew J Lee^1^, Thomas Pinkney^1^ Sheraz R Markar^2, 3^

1. Department of Applied Health Sciences, School of Health Sciences, College of Medicine and Health, University of Birmingham, Birmingham, United Kingdom
2. Nuffield Department of Surgery, University of Oxford, Oxford, UK.
3. Department of Surgery, Churchill Hospital, Oxford University Hospitals NHS Trust, Oxford, UK.
4. Academic Department of Abdominal Wall Surgery, North Devon District Hospital, Royal Devon University Healthcare NHS Foundation Trust, Barnstaple, United Kingdom.
5. Department of Clinical and Biomedical Sciences, University of Exeter Medical School, Exeter, United Kingdom.
6. NIHR Exeter Biomedical Research Centre, Exeter, United Kingdom

**Corresponding author:** Mr Sivesh Kamarajah, NIHR Doctoral Fellow, Department of Applied Health Sciences, School of Health Sciences, University of Birmingham, Birmingham B15 2TH, UK. Email: [s.k.kamarajah@bham.ac.uk](mailto:s.k.kamarajah@bham.ac.uk)

**Number of words:** 3,837 words **Number of pages:** 10

**Number of tables:** 2

**Number of figures:** 3

**Abstract word count:** 415 words

**Keywords:** GLP1; safety; surgical systems; evidence synthesis

**Short title:** Safety of preoperative GLP-1

**Data sharing:** Data sharing requests will be considered by the writing group upon written request to the corresponding authors.

**Conflict of interest:** There are no conflicts of interest to declare.

Table of Contents

[**Table S1.** PRISMA checklist for the systematic review 3](#_Toc195605536)

[**Table S2.** Summary of search terms for the systematic review. 6](#_Toc195605537)

[**Table S3.** Summary of data extraction sheet 7](#_Toc195605538)

[**Table S4.** Risk of bias assessment for randomised controlled trials using the Cochrane Risk of Bias-2 tool. 8](#_Toc195605539)

[**Table S5.** Risk of bias assessment for non-randomised, comparative studies using ROBINS-I tool 9](#_Toc195605540)

[**Table S6.** Risk of bias assessment for non-randomised, non-comparative studies using Newcastle Ottawa Scale 10](#_Toc195605541)

[**Figure S1.** Publication bias performed for studies included in the meta-analysis of the primary outcome of postoperative complications. 11](#_Toc195605542)

[**Table S7.** GRADE evidence profile for the use of preoperative GLP-1 in patients undergoing surgery. 12](#_Toc195605543)

[**Table S8.** Adverse events of preoperative GLP-1 in studies reporting them 13](#_Toc195605544)

[**Appendix A.** Risk of bias assessment of the included studies 14](#_Toc195605545)

## **Table S1.** PRISMA checklist for the systematic review

| **Section and Topic** | **Item #** | **Checklist item** | **Location where item is reported** |
| --- | --- | --- | --- |
| **TITLE** | | |  |
| Title | 1 | Identify the report as a systematic review. | 1 |
| **ABSTRACT** | | |  |
| Abstract | 2 | See the PRISMA 2020 for Abstracts checklist. | 2-3 |
| **INTRODUCTION** | | |  |
| Rationale | 3 | Describe the rationale for the review in the context of existing knowledge. | 4 |
| Objectives | 4 | Provide an explicit statement of the objective(s) or question(s) the review addresses. | 4 |
| **METHODS** | | |  |
| Eligibility criteria | 5 | Specify the inclusion and exclusion criteria for the review and how studies were grouped for the syntheses. | 5 |
| Information sources | 6 | Specify all databases, registers, websites, organisations, reference lists and other sources searched or consulted to identify studies. Specify the date when each source was last searched or consulted. | 5 |
| Search strategy | 7 | Present the full search strategies for all databases, registers and websites, including any filters and limits used. | 5 |
| Selection process | 8 | Specify the methods used to decide whether a study met the inclusion criteria of the review, including how many reviewers screened each record and each report retrieved, whether they worked independently, and if applicable, details of automation tools used in the process. | 5-6 |
| Data collection process | 9 | Specify the methods used to collect data from reports, including how many reviewers collected data from each report, whether they worked independently, any processes for obtaining or confirming data from study investigators, and if applicable, details of automation tools used in the process. | 6 |
| Data items | 10a | List and define all outcomes for which data were sought. Specify whether all results that were compatible with each outcome domain in each study were sought (e.g. for all measures, time points, analyses), and if not, the methods used to decide which results to collect. | 6-7 |
|  | 10b | List and define all other variables for which data were sought (e.g. participant and intervention characteristics, funding sources). Describe any assumptions made about any missing or unclear information. | 6-7 |
| Study risk of bias assessment | 11 | Specify the methods used to assess risk of bias in the included studies, including details of the tool(s) used, how many reviewers assessed each study and whether they worked independently, and if applicable, details of automation tools used in the process. | 7 |
| Effect measures | 12 | Specify for each outcome the effect measure(s) (e.g. risk ratio, mean difference) used in the synthesis or presentation of results. | 7-9 |
| Synthesis methods | 13a | Describe the processes used to decide which studies were eligible for each synthesis (e.g. tabulating the study intervention characteristics and comparing against the planned groups for each synthesis (item #5)). | 8-9 |
|  | 13b | Describe any methods required to prepare the data for presentation or synthesis, such as handling of missing summary statistics, or data conversions. | 8-9 |
|  | 13c | Describe any methods used to tabulate or visually display results of individual studies and syntheses. | 8-9 |
|  | 13d | Describe any methods used to synthesize results and provide a rationale for the choice(s). If meta-analysis was performed, describe the model(s), method(s) to identify the presence and extent of statistical heterogeneity, and software package(s) used. | 8-9 |
|  | 13e | Describe any methods used to explore possible causes of heterogeneity among study results (e.g. subgroup analysis, meta-regression). | 8-9 |
|  | 13f | Describe any sensitivity analyses conducted to assess robustness of the synthesized results. | 8-9 |
| Reporting bias assessment | 14 | Describe any methods used to assess risk of bias due to missing results in a synthesis (arising from reporting biases). | 8-9 |
| Certainty assessment | 15 | Describe any methods used to assess certainty (or confidence) in the body of evidence for an outcome. | 8-9 |
| **RESULTS** | | |  |
| Study selection | 16a | Describe the results of the search and selection process, from the number of records identified in the search to the number of studies included in the review, ideally using a flow diagram. | 10-11 |
|  | 16b | Cite studies that might appear to meet the inclusion criteria, but which were excluded, and explain why they were excluded. | 10-11 |
| Study characteristics | 17 | Cite each included study and present its characteristics. | 10-11 |
| Risk of bias in studies | 18 | Present assessments of risk of bias for each included study. | 10-11 |
| Results of individual studies | 19 | For all outcomes, present, for each study: (a) summary statistics for each group (where appropriate) and (b) an effect estimate and its precision (e.g. confidence/credible interval), ideally using structured tables or plots. | 10-11 |
| Results of syntheses | 20a | For each synthesis, briefly summarise the characteristics and risk of bias among contributing studies. | 10-11 |
|  | 20b | Present results of all statistical syntheses conducted. If meta-analysis was done, present for each the summary estimate and its precision (e.g. confidence/credible interval) and measures of statistical heterogeneity. If comparing groups, describe the direction of the effect. | 10-11 |
|  | 20c | Present results of all investigations of possible causes of heterogeneity among study results. | 10-11 |
|  | 20d | Present results of all sensitivity analyses conducted to assess the robustness of the synthesized results. | 10-11 |
| Reporting biases | 21 | Present assessments of risk of bias due to missing results (arising from reporting biases) for each synthesis assessed. | 10-11 |
| Certainty of evidence | 22 | Present assessments of certainty (or confidence) in the body of evidence for each outcome assessed. | 10-11 |
| **DISCUSSION** | | |  |
| Discussion | 23a | Provide a general interpretation of the results in the context of other evidence. | 12-14 |
|  | 23b | Discuss any limitations of the evidence included in the review. | 14 |
|  | 23c | Discuss any limitations of the review processes used. | 14 |
|  | 23d | Discuss implications of the results for practice, policy, and future research. | 13-14 |
| **OTHER INFORMATION** | | |  |
| Registration and protocol | 24a | Provide registration information for the review, including register name and registration number, or state that the review was not registered. | 2 |
|  | 24b | Indicate where the review protocol can be accessed, or state that a protocol was not prepared. | 6 |
|  | 24c | Describe and explain any amendments to information provided at registration or in the protocol. | 6 |
| Support | 25 | Describe sources of financial or non-financial support for the review, and the role of the funders or sponsors in the review. | 2 |
| Competing interests | 26 | Declare any competing interests of review authors. | 2 |
| Availability of data, code and other materials | 27 | Report which of the following are publicly available and where they can be found: template data collection forms; data extracted from included studies; data used for all analyses; analytic code; any other materials used in the review. | 2 |

## **Table S2.** Summary of search terms for the systematic review.

| 1 | exp Glucagon-Like Peptide 1/ or exp Glucagon-Like Peptide-1 Receptor/ | 15578 |
| --- | --- | --- |
| 2 | (tirzepatide or semaglutide or liraglutide or Retatrutide).ti,ab. | 6147 |
| 3 | (glucagon-like peptide 1 or glucagon like peptide 1 or glp 1 or glucagon-like peptide-1).ab,ti. | 22222 |
| 4 | 1 or 2 or 3 | 26168 |
| 5 | (randomized controlled trial or controlled clinical trial or pragmatic clinical trial).pt. or (randomis* or randomiz* or randomly).ti,ab. or (trial or multicenter or multi center or multicentre or multi centre).ti. or clinical trials as topic.sh. or randomly.ab. or (random$ or placebo$ or single blind$ or double blind$ or triple blind$).ti,ab. or random.ti,ab. | 2114846 |
| 6 | (((exp animals/ not humans.sh.) or (animals not humans).sh. or ((comment or editorial or news or meta-analysis or practice-guideline or review or letter) not "randomized controlled trial").pt. or (random sampl$ or random digit$ or random effect$ or random survey or random regression).ti,ab.) not "randomized controlled trial".pt.) or (systematic review or literature review).ti. or comment on.cm. or cohort.ti. or (propensity or retrospective).ti,ab. | 12172599 |
| 7 | 5 not 6 | 1439890 |
| 8 | 4 and 7 | 3487 |
| 9 | (surgery or surgical or resection).ti,ab. | 2390580 |
| 10 | 4 and 9 | 1786 |

## **Table S3.** Summary of data extraction sheet

| **Category** | **Definition** |
| --- | --- |
| **Study characteristics** | |
| Study name | Includes author name and year |
| Study year | Study starts and end date |
| Country income | High income / Low- or middle-income defined according to World Bank group |
| Country | This is defined as the countries in which the study was delivered. |
| **Patient characteristics** | |
| Age | This refers to the mean or median age of the cohort in the study, across with and without preoperative GLP-1. |
| Gender, female | The proportion of female patients in the study, across those with and without preoperative GLP-1. |
| Body mass index, kg/m^2^ | The mean or median body max index of patients in the study, across those with and without preoperative GLP-1. |
| Diabetes | The proportion of patients with diabetes in the study, across those with and without preoperative GLP-1. |
| Duration of preoperative GLP-1 | This refers to the duration of GLP-1 given before surgery. |
| **Key outcomes** | |
| Postoperative complications | This defined as complications occurring from surgery up to 90-days, defined according to the Clavien-Dindo classification. |
| Weight loss | Any weight loss before surgery from the preoperative use of GLP-1 |

## **Table S4.** Risk of bias assessment for randomised controlled trials using the Cochrane Risk of Bias-2 tool.

| **Study name** | **Domain 1** | **Domain 2** | **Domain 3** | **Domain 4** | **Domain 5** | **Overall** |
| --- | --- | --- | --- | --- | --- | --- |
| Polderman 2018 | Low | Some | Low | Low | Low | Some concerns |
| Hulst 2020 | Low | Low | Low | Low | Low | Low |

## **Table S5.** Risk of bias assessment for non-randomised, comparative studies using ROBINS-I tool

| **Study name** | **Domain 1** | **Domain 2** | **Domain 3** | **Domain 4** | **Domain 5** | **Domain 6** | **Domain 7** | **Overall** |
| --- | --- | --- | --- | --- | --- | --- | --- | --- |
| Wood 2016 | Moderate | Moderate | Low | Low | Moderate | Low | Moderate | Moderate |
| Ilanga 2023 | Critical | Low | Low | Low | Low | Low | Critical | Critical |
| Martines 2023 | Serious | Serious | Low | Moderate | Low | Moderate | Moderate | Serious |
| Dixit 2024 | Moderate | Low | Moderate | Low | Low | Low | Low | Moderate |
| Magruder 2024 | Serious | Moderate | Low | Low | Moderate | Low | Moderate | Serious |
| Martines 2024 | Moderate | Moderate | Low | Low | Low | Moderate | Moderate | Moderate |
| Oosterom-Eijmael 2024 | Low | Low | Low | Low | Low | Low | Low | Low |
| Spurzem 2024 | Moderate | Low | Low | Low | Low | Low | Low | Low |
| Welk 2024 | Moderate | Low | Low | Low | Low | Low | Low | Moderate |
| AbuHasan 2025 | Critical | Moderate | Low | Low | Low | Low | Serious | Critical |
| Sen 2025 | Low | Moderate | Low | Low | Low | Low | Low | Low |
| Kim 2025 | Low | Low | Low | Low | Low | Low | Low | Low |
| Mathur 2025 | Serious | Moderate | Moderate | Low | Moderate | Low | Moderate | Serious |
| Rayman 2025 | Critical | Serious | Moderate | Low | Serious | Critical | Moderate | Critical |
| Seddio 2025 | Low | Low | Low | Low | Low | Low | Low | Low |
| Aschen 2025 | Moderate | Low | Moderate | Low | Low | Moderate | Low | Moderate |

## **Table S6.** Risk of bias assessment for non-randomised, non-comparative studies using Newcastle Ottawa Scale

| **Study name** | **Domain 1** | **Domain 2** | **Domain 3** | **Domain 4** | **Domain 5** | **Domain 6** | **Domain 7** | **Domain 8** | **Domain 9** | **Overall** |
| --- | --- | --- | --- | --- | --- | --- | --- | --- | --- | --- |
| Tang 2017 | Yes | No | Yes | Yes | Yes | No | Yes | Yes | Yes | Moderate-to-low |
| Rye 2018 | Yes | No | Yes | Yes | Yes | No | Yes | Yes | Yes | Moderate-to-Low |
| Rubio-Herrera 2023 | Yes | No | Yes | Yes | Yes | No | Yes | Yes | Yes | Moderate-to-Low |
| Gonzalez 2024 | Yes | No | Yes | Yes | Yes | No | Yes | Yes | Yes | Moderate-to-Low |
| Munoz 2024 | Yes | No | Yes | Yes | No | No | Yes | Yes | Yes | Moderate |

##
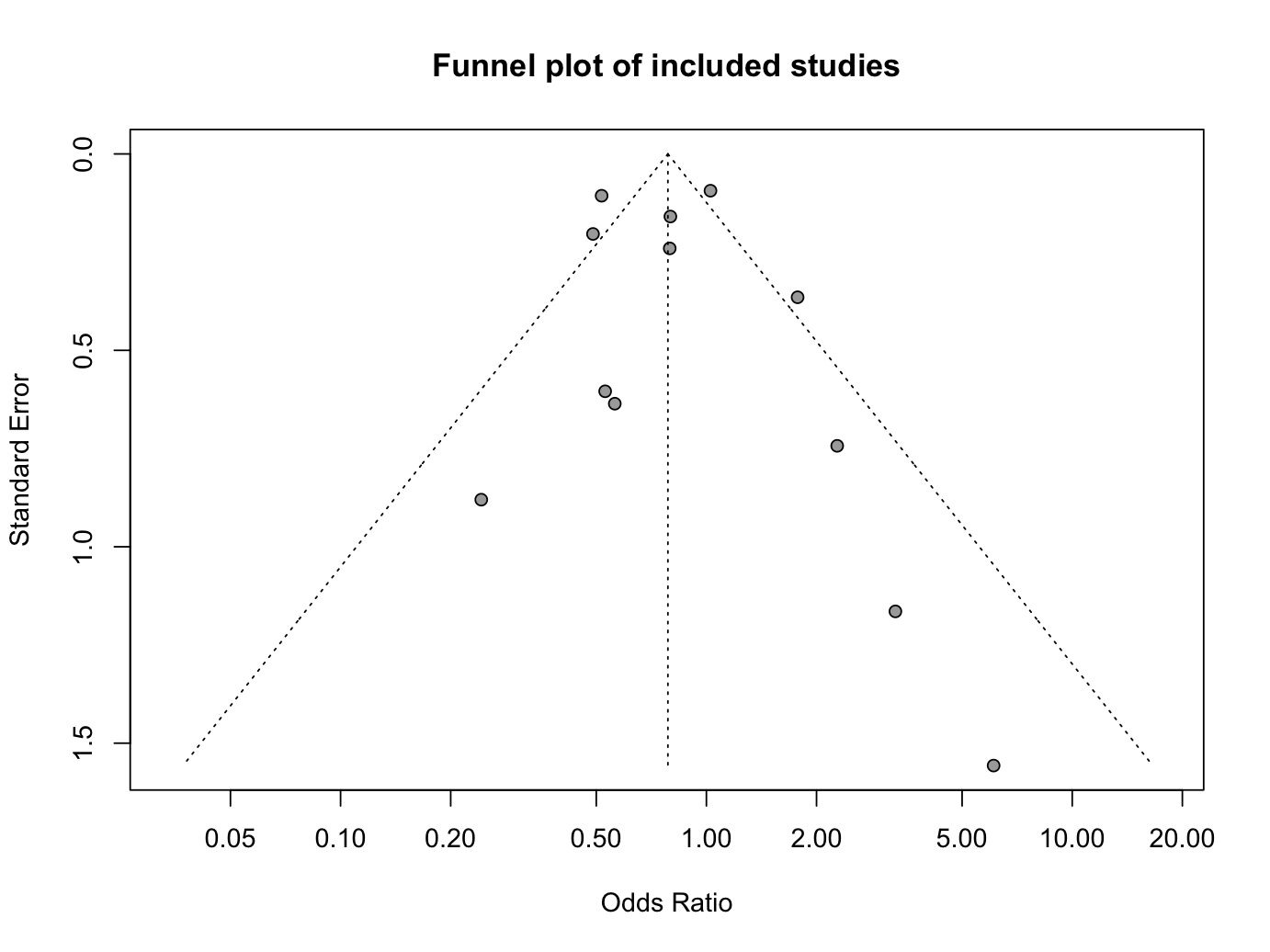
**Figure S1.** Publication bias performed for studies included in the meta-analysis of the primary outcome of postoperative complications.

## **Table S7.** GRADE evidence profile for the use of preoperative GLP-1 in patients undergoing surgery.

| **Outcome** | **Participants (Studies)** | **Study design** | **Risk of bias** | **Inconsistency** | **Indirectness** | **Imprecision** | **Publication bias** | **Overall certainty of evidence** | **Summary** |
| --- | --- | --- | --- | --- | --- | --- | --- | --- | --- |
| 90-days postoperative complications | 43,302  (12 studies) | 1 RCT, 11 NRS | Serious | Serious | Not serious | Serious | Not serious | Very Low | Pooled analysis suggests GLP-1RA may reduce risk, but estimate is highly uncertain due to design limitations and unexplained heterogeneity |

## **Table S8.** Adverse events of preoperative GLP-1 in studies reporting them

| **Study name** | **Timing** | **Definition** | **Events** |
| --- | --- | --- | --- |
| Polderman 2018 | 30 days after surgery | Hypoglycaemia, nausea (scored on an 11-point NRS with severe nausea defined as NRS > 4), hypo- and hyperkalaemia. | 21 (47.8%) |
| Rye 2018 | 16 weeks; 28 weeks | Nausea | 11 (37.9%) |
|  |  | Headache | 11 (37.9%) |
|  |  | Gastroesophageal reflux | 10 (34.5%) |
|  |  | Constipation | 9 (31.0%) |
|  |  | diarrhoea (9 patients, 31.0%), | 9 (31.0%) |
|  |  | Vomiting | 4 (13.8%) |
|  |  | Dizziness | 4 (13.8%) |
|  |  | Fatigue | 3 (10.3%) |
| Hulst 2020 | 30 days after surgery | Monitoring for hypoglycaemia (mild and severe), nausea and vomiting, mortality, and postoperative complications. | 36 (26%) |
| Ilanga 2023 | Not reported | Nausea, emesis, diarrhoea, hypoglycaemia, injection site reactions, and pancreatitis. | 0 (0%) |
| Rubio-Herrera 2023 | 6 weeks | Nausea | 33 (32.4%) |
|  |  | Vomiting | 8 (7.4%) |
|  |  | Constipation | 16 (23.9%) |
|  |  | Diarrhoea | 7 (18.6%) |
| Gonzalez 2024 | Not reported | Nausea | 3 (15.7%) |
|  |  | Diarrhoea | 3 (15.7%) |
|  |  | Vomiting | 2 (10.5%) |
|  |  | Constipation | 2 (10.5%) |
| Martines 2024 | Not reported | Gastrointestinal symptoms such as nausea, diarrhoea, vomiting, and abdominal fullness, as well as more severe conditions like pancreatitis. | 0 (0%) |
| Munoz 2024 | 30 days after surgery | Any events such as constipation, burping and nausea, and rash. | 22 (59%) |
| Welk 2024 | 14-days after surgery | Aspiration pneumonia | 5 (0.1%) |
| Sen 2025 | Not reported | Residual gastric content | 35 (56.5%) |
| Rayman 2025 | Not reported | Any | 179 (65.1%) |
|  |  | Gastrointestinal symptoms such as nausea or vomiting, diarrhoea, abdominal pain, bloating, hiccups, and heartburn | 159 (57.8%) |
|  |  | Neuropsychiatric defined as dizziness, headache, tiredness or weakness and depressed mood | 43 (9.9%) |
|  |  | Cardiac | 2 (0.5%) |
|  |  | Endocrine | 5 (1.2%) |

## **Appendix A.** Risk of bias assessment of the included studies

Methodological quality of all included studies was independently evaluated by two reviewers (SKK and NG) using validated, design‐specific tools. For each study, the review process was conducted in duplicate, with both reviewers assessing each study independently. Discrepancies between reviewers were resolved through discussion, and when consensus could not be reached, a third independent reviewer (SRM) was consulted. All quality assessments were repeated at a separate time point to ensure reliability and consistency. For randomised studies, we assessed risk of bias using the Cochrane Risk of Bias 2 (RoB-2) tool. This tool evaluates bias across five domains: (i) bias arising from the randomisation process; (ii) bias due to deviations from intended interventions; (iii) bias due to missing outcome data; (iv) bias in the measurement of the outcome; and (v) bias in the selection of the reported result. For non-randomised, we used the Cochrane ROBINS-I tool, consisting of seven domains. These domains are: (i) bias due to confounding; (ii) bias in the selection of participants into the study; (iii) bias in the classification of interventions; (iv) bias due to deviations from intended interventions; (v) bias due to missing data; (vi) bias in measurement of outcomes; and (vii) bias in selection of the reported result. For studies without a comparison group, we used the Newcastle-Ottawa Scale (NOS) to assess study quality and risk of bias based on selection, comparability, and outcome assessment.
